# HEARTS in the Americas: Targeting Health System Change to Improve Population Hypertension Control

**DOI:** 10.1007/s11906-023-01286-w

**Published:** 2023-12-02

**Authors:** Pedro Ordunez, Norm R. C. Campbell, Donald J. DiPette, Marc G. Jaffe, Andres Rosende, Ramon Martinez, Angelo Gamarra, Cintia Lombardi, Natalia Parra, Libardo Rodriguez, Yenny Rodriguez, Jeffrey Brettler

**Affiliations:** 1 Department of Non-Communicable Diseases and Mental Health, Pan American Health Organization, Washington, DC USA; 2 Department of Medicine, Libin Cardiovascular Institute, The University of Calgary, Calgary, AB T2N 1N4 Canada; 3 University of South Carolina and University of South Carolina School of Medicine, Columbia, SC USA; 4 Department of Endocrinology, The Permanente Medical Group, Kaiser San Francisco Medical Center, San Francisco, CA USA; 5 Southern California Permanente Medical Group, Department of Health Systems Science, Regional Hypertension Program, Kaiser Permanente Bernard J. Tyson School of Medicine, Pasadena, USA

**Keywords:** Hypertension, Cardiovascular diseases, Primary health care, Public health, Americas

## Abstract

**Purpose of Review:**

HEARTS in the Americas is the regional adaptation of Global Hearts, the World Health Organization initiative for cardiovascular disease (CVD) prevention and control. Its overarching goal is to drive health services to change managerial and clinical practice in primary care settings to improve hypertension control and CVD risk management. This review describes the HEARTS in the Americas initiative. First, the regional epidemiological situation of CVD mortality and population hypertension control trends are summarized; then the rationale for its main intervention components: the primary care-oriented management system and the HEARTS Clinical Pathway are described. Finally, the key factors for accelerating the expansion of HEARTS are examined: medicines, team-based care, and a system for monitoring and evaluation.

**Recent Findings:**

Thus far, 33 countries in Latin America and the Caribbean have committed to integrating this program across their primary healthcare network by 2025. The increase in hypertension coverage and control in primary health care settings compared with the traditional model is promising and confirms that the interventions under the HEARTS umbrella are feasible and acceptable to communities, patients, providers, decision-makers, and funders. This review highlights some cases of successful implementation.

**Summary:**

Scaling up effective treatment for hypertension and optimization of CVD risk management is a pragmatic way to accelerate the reduction of CVD mortality while strengthening primary healthcare systems to respond effectively, with quality, and equitably, to the challenge of non-communicable diseases, not only in low-middle income countries but in all communities globally.

## Introduction

HEARTS in the Americas is the regional adaptation of Global Hearts, the World Health Organization (WHO) initiative for cardiovascular disease (CVD) prevention and control [1, 2]. HEARTS in the Americas is coordinated by national Ministries of Health with the collaboration of local stakeholders and technical support of the Pan American Health Organization (PAHO) and other partners such as the U.S Centers for Disease Control and Prevention (CDC), Resolve to Save Lives, and World Hypertension League. The overarching goal of HEARTS in the Americas is to drive health services to change managerial and clinical practice in primary care settings to improve hypertension control and CVD risk management. Thus far, 33 countries in Latin America and the Caribbean (LAC) have committed to integrating this program across their primary healthcare (PHC) network by 2025. Over 3000 PHC facilities serving more than 4 million individuals in treatment are applying this model of care [3].

With 2 million deaths annually, CVD remains the deadliest disease in all countries of the Americas [4•]. CVD is the main cause of premature mortality, reducing the population’s life expectancy regionwide, and is a major cause of disability and socioeconomic disparity [5]. High systolic blood pressure (SBP) is the main modifiable risk factor for CVD. Hypertension (≥ 140/90 mmHg) affects more than a third of adults in this region [6], a number that is much higher if those at high CVD risk and SBP ≥ 130 mmHg are included. Suboptimal blood pressure (BP) control is the leading population-attributable risk factor for CVD including hemorrhagic stroke (population attributable fraction = 58%), ischemic stroke (50%), and ischemic heart disease (IHD) (55%) [7].

In LAC in 2019, a year before the COVID-19 pandemic, 37% of people with hypertension had not been diagnosed, 15% of those diagnosed were not receiving treatment, and 47% of those receiving treatment did not have their BP controlled (< 140/90 mm Hg) [8]. Likewise, data show low rates of use of known effective CVD secondary prevention medications (blood pressure-lowering drugs, statins, and aspirin) in individuals with self-reported CVD [9]. Suboptimal CVD management is not exclusive to low-middle-income countries (LMIC). For instance, declining levels of hypertension control have been reported in the USA [10] and Canada [11].

HEARTS in the Americas emerged as a response to the Sustainable Development Goal of reducing premature mortality from non-communicable diseases (NCD) by 2030 by one-third. It was based on the “Global Standardized Hypertension Treatment Project,” an endeavor between the CDC and PAHO [12]. Then, it developed in parallel with the WHO Global Hearts [2] and has been significantly influenced by Resolve to Save Lives [13], the World Hypertension League [14], and the World Heart Federation [15, 16]. HEARTS in the Americas has been inspired by and modeled after successful hypertension population control programs in North America, particularly The Canadian Hypertension Education Program [17] and the Kaiser Permanente hypertension program in California, USA [18].

Implementation of hypertension programs has been largely limited to high-income countries. This manuscript reviews a major initiative crafted for LMIC where the associated global burden attributable to hypertension is overwhelming. This review describes the HEARTS in the Americas initiative. First, the regional epidemiological situation of CVD mortality and population hypertension control trends is summarized to justify an intervention of this nature; then the rationale for its main intervention components: the primary care-oriented management system and the HEARTS Clinical Pathway are described. Finally, the key factors for accelerating the expansion of HEARTS are summarized. The manuscript highlights some cases of successful implementation that provide the potential of HEARTS to improve population-wide hypertension control and reduce the burden of CVD in the Americas.

## Regional Epidemiological Situation of CVD Mortality and Hypertension Control

### Cardiovascular Diseases, Ischemic Heart Disease and Stroke Mortality

Over the last three decades (1990–2019), the Americas have experienced a substantial reduction in CVD mortality. Regional age-standardized CVD mortality rate fell by 42% reaching 162.3 deaths/100,000 population in 2019. While the USA and Canada reached 157.0 and 107.9 deaths/100,000, respectively in 2019, Caribbean countries have the highest CVD mortality rates in the region. IHD and stroke still rank as the first and second leading regional causes of death in 2019 [19, 20]. Socioeconomic growth, better access to health services, advances in treatments, improved hypertension control, and a notable decrease in tobacco use explain this trend [21].

**Fig. 1:**
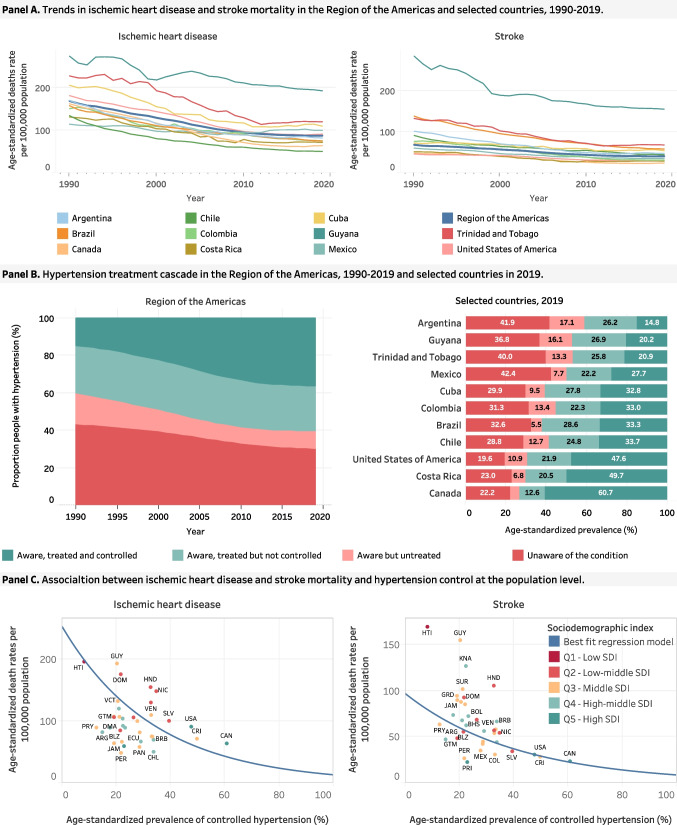
CVD mortality and hypertension control in the Americas 1990–2019. Figure created by the authors for this paper. In graphs from C, dots represent countries, and they are identified using their ISO-3166–1 three-letter (3-alpha) codes.|

The rate of decline in CVD mortality from 1990 to 2019 differed across countries, slowing by half between 2010 and 2019, dropping from a 2% to a 1% annual reduction regionwide [4•]. The USA is a notable example of this trend, which is also observed in other countries like Argentina, Brazil, Colombia, Canada, Cuba, Ecuador, and Trinidad and Tobago (Fig. 1A). Several factors could account for this, including economic slowdown, persistent socioeconomic disparities, a slower rate of decline in tobacco use, increased obesity, and diabetes rates, and low rates of hypertension control. Additional data on the burden of CVD, IHD, and stroke in the Americas and by country are available in the PAHO ENLACE data portal, https://www.paho.org/en/enlace/cardiovascular-disease-burden.

### High Blood Pressure and Hypertension Treatment Cascade

Figure 1B shows the hypertension treatment cascade, regional trends from 1990 to 2019 and levels for selected countries for 2019. The prevalence of detection/awareness, treatment, and control in the region improved from 1990 to 2019. Overall, the percentage of population hypertension control improved approximately twofold in this period. There were significant improvements in hypertension control among those treated, a slight improvement in the percentage of treated among those detected, and very little improvement in detection. There is huge variation between countries. Further data on the prevalence of hypertension, and hypertension awareness, treatment, and control in the Americas are available in the PAHO ENLACE data portal https://www.paho.org/en/enlace/hypertension.

In summary, the Region of the Americas, with a prevalence of hypertension (≥ 140/90 mmHg or taking medications) around 35%, like those of other WHO regions, has the highest population hypertension control rates globally [22]. For instance, among all persons with hypertension, the control rate is 10% higher in the Americas than in Europe (36% vs. 26%), and the Americas has a higher detection rate (about 70% vs. 66%), and a better treatment rate (60% vs. 53%). Canada and the USA are among the nations with the highest hypertension control rates in the world and have had a substantial influence on these regional results. However, the situation of LAC is quite different, even though some countries such as Costa Rica show strong hypertension control figures.

### Association Between IHD and Stroke Mortality and Population Hypertension Control

In the Americas, consistent with clinical trials and observational studies, there is a strong inverse association between the levels of IHD and stroke mortality and the population hypertension control (Fig. 1C). Countries with the highest levels of population hypertension control tend to have low IHD and stroke mortality levels. This ecologic study with data from 36 countries and territories of the Americas from 1990 to 2019, found that one unit increase in population hypertension control was associated with a reduction of 2.9% deaths/100,000 population caused by IHD and 2.4% deaths/100,000 due to stroke [4•]. Indeed, if the Americas improved population-based hypertension control from the current level of 36% to a target of 50%, over 400,000 CVD deaths could be avoided. Furthermore, if secondary CVD prevention efforts were expanded, many more deaths could be averted [23].

To help make these projections, PAHO developed the CVD:HTN EstimaTool, which is an interactive tool to estimate the number of IHD and stroke deaths that could be averted in a location or population group by improving the population hypertension control from a current to a target level in a given number of years. It is available at https://www.paho.org/en/enlace/tool-estimate-impact-population-hypertension-control-cvd-mortality.

## The Strengthening of a Primary Care-oriented Management System

### The Political Will to Prioritize Hypertension and Social Mobilization in Action

Hypertension is a massive population health problem. Its prevention and control demand a life-course strategy [24] and a whole-of-society approach to health through multisectoral policies and actions; empowering people and communities; and primary care as the core of integrated health services [25]. Therefore, hypertension programs require strong political will, accountable coordination mechanisms at every level of the health system, stakeholder engagement, strong community activism, health-financial protection mechanisms in place, budget allocation, and an effective primary health care (PHC) approach that has the capacity for the whole population.

Governments of the region, supported by PAHO and other partners, are progressing in the implementation of multisectoral policies that lead to tobacco control [26], salt reduction [27], the elimination of industrially produced trans-fat [28], and the application of front-of-package nutrition labeling [29]. In addition, LAC civil society is working to prioritize hypertension control on the political agenda by advocating for resources, strengthening community awareness, and creating social demand for action [30]. Likewise, the Inter-American Society of Cardiology has adopted a strong position in supporting the WHO guidelines for the pharmacological treatment of hypertension [31, 32••] through the implementation of HEARTS across the region [33].

Hypertension is one of the most common diagnoses managed in the primary care setting and, in many cases, is the point of entry into the care system. In addition, it has enormous potential to shape the management of other NCDs at this level of care. Therefore, HEARTS in the Americas has called for prioritizing this initiative in the health ecosystem oriented to PHC [3]. This requires a system that supports the achievement of universal coverage, equity, and access to quality services and is acceptable to the population in a variety of social and cultural settings [25].

### Building an Effective and Evidence-based Primary Care Delivery System

Successful hypertension programs share many attributes which are all relevant and interconnected (Box 1) [34]. Moreover, a prerequisite for building an effective PHC system is to implement practices and programs based on evidence. This, among other factors, allows for gaining the trust and support of clinicians, the public/patients, and all interested parties. Indeed, the 2021 WHO Guideline for Pharmacological Treatment of Hypertension in Adults [31, 32••, 35] is the clinical foundation for HEARTS in the Americas. It is guided by best practices and is transparent, inclusive, multicultural, and implementation-oriented. These attributes facilitate rapid adherence by countries, organizations, health systems, and clinicians, and reduce the risk of conflicts of interest. In addition, the recommendations of the WHO hypertension guideline are consistent with most major hypertension guidelines worldwide [36, 37] (Table 1).

**Table 1:** HEARTS Clinical Pathway. Similarities and differences with selected hypertension guidelines

Recommendation Area	HEARTS in the Americas Clinical Pathway	Major hypertension guidelines		
		2021 WHO	2017 ACC/AHA	2023 ESH
Number of pages*	1	29	63	142
N° recommendations	8 drivers	8	106	273
Implementation oriented	++++	+++	+	+ / −
Target patient group	Most hypertensive patients	Most hypertensive patients	All hypertensive patients (secondary hypertension, pregnancy, comorbidities, and others)	All hypertensive patients (secondary hypertension, pregnancy, comorbidities, and others)
Target healthcare provider group	Primary care including non-physician healthcare workers	Primary care including non-physician healthcare workers	Specialized and physician-centered healthcare	Specialized and physician-centered healthcare
Diagnosis and classification of HTN	Simple 2 categories (according to CVD risk)	Simple 2 categories (according to CVD risk)	Simple 2 categories (according to comorbidities)	Complex 5 categories
Patient evaluation	Simple	Simple	Complex	Complex
CVD risk estimation	Simple and pragmatic approach: 3 categories CVD risk assessment at or after the initiation of pharmacological treatment for hypertension, but only where this is feasible and does not delay treatment	Simple and pragmatic approach: 3 categories CVD risk assessment at or after the initiation of pharmacological treatment for hypertension, but only where this is feasible and does not delay treatment	Complex: multiple categories	Complex: multiple categories
BP thresholds for antihypertensive drug therapy	BP ≥ 140/90 in all patients SBP ≥ 130 in patients with existing CVD, high-calculated CVD risk, diabetes mellitus, or chronic kidney disease	BP ≥ 140/ ≥ 90 in all patients SBP 130–139 in patients with existing CVD, high-calculated CVD risk, diabetes mellitus, or chronic kidney disease	BP ≥ 140/90 in all patients BP ≥ 130/80 in high-CVD-risk patients	BP ≥ 140/90 in all patients < 80 years SBP ≥ 160 in patients ≥ 80 years BP ≥ 130/80 in patients with CVD
BP control targets	BP < 140/90 in all patients SBP < 130 in patients with existing CVD, high-calculated CVD risk, diabetes mellitus, or chronic kidney disease	BP < 140/90 in all patients SBP < 130 in patients with existing CVD, high-calculated CVD risk, diabetes mellitus, or chronic kidney disease	BP < 130/80 in all patients	BP < 140/90 in all patients BP 120–129/70–79 if tolerated
Healthy lifestyle recommendations	Yes	Not applicable	Yes	Yes
Antihypertensive drug therapy	Using a protocol with specific drugs and doses Combined therapy in all patients, preferably in FDC	Using a protocol with specific drugs and doses Combined therapy in all patients, preferably in FDC	Drug therapy under medical decision Combined therapy in most patients, preferably in FDC	Drug therapy under medical decision Combined therapy in most patients, preferably in FDC
Complementary medication	Aspirin + high-intensity therapy with statins in patients with CVD Moderate-intensity therapy with statins in patients with high-calculated CVD risk, diabetes mellitus, or chronic kidney disease	Not mentioned	Not mentioned	Aspirin in patients with CVD + Statins to achieve the LDL goal in high-CVD risk patients
Follow-up intervals	Monthly until reaching BP control Every 6 months if BP is controlled Every 3 months in patients with existing CVD, high-calculated CVD risk, diabetes mellitus, or chronic kidney disease if BP is controlled	Monthly until reaching BP control Every 3–6 months if BP is controlled	Monthly until reaching BP control Every 3–6 months if BP is controlled	Monthly during the first 3 months after treatment initiation Non-specific follow-up intervals if BP is controlled
Vaccination	COVID-19 in all patients Influenza and Pneumococcus in patients with existing CVD, high-calculated CVD risk, diabetes mellitus, or chronic kidney disease	Not mentioned	Not mentioned	COVID-19 in all patients

Table created by authors for this paper

Box 1. Key Attributes of Successful Hypertension Programs

- Bold leadership with a sense of urgency to lead change, particularly in PHC settings.
- Basic infrastructure to build trust in the community it serves, including free and easy access at the point of care.
- A core set of quality and affordable medicines for hypertension, statins, and diabetes medicines, including those for kidney protection.
- Clinically validated automated blood pressure devices.
- Enough workforce, motivated, adequately compensated, and trained for team-based care.
- Timely, accurate, reliable, and efficient mechanisms for hypertension detection, diagnosis, and follow-up.
- A clinical pathway with a simple and standardized treatment protocol.
- A functional health information system for clinical monitoring and performance evaluation

## Institutionalization and Systematic Implementation of the HEARTS Clinical Pathway

### HEARTS Hypertension Control Drivers

Suboptimal population hypertension control can no longer be primarily attributed to patient responsibility or clinicians. This mindset is at the root of the long-standing failure to adopt practical and effective solutions to improve population hypertension control. Indeed, there are multiple barriers to diagnosis, treatment, and continuity of care, most related to the healthcare delivery system rather than patient or provider behavior [13]. Consequently, hypertension programs require identifying barriers to access, selecting interventions to overcome bottlenecks, and optimizing the delivery processes involved in the hypertension treatment cascade to improve the health system’s performance [3].

HEARTS in the Americas appointed an innovation group (IG) consisting of a multidisciplinary group of experts, with in-depth knowledge of the field, from the first 12 implementing countries, to review the recommendations of the major hypertension treatment guidelines. The key questions were not what the evidence is but how to implement the recommendations. Additionally, the IG emphasized reviewing hypertension programs in high-performance health systems to understand what and how they did to make things work well and be sustainable [40].

As a result, following the five main domains of care delivery, i.e., diagnosis, treatment, continuity of care, delivery system, and monitoring and evaluation, the IG identified eight critical and interdependent interventions that, applied systematically, led to substantial and sustained improvements in hypertension control in relatively large health systems. These key drivers of hypertension control resulted in 17 very specific implementable actions (Table 2).

**Table 2:** HEARTS in the Americas. Key drivers for hypertension control (Adapted from Ref 41)

Areas	Drivers	Interventions
Diagnosis	1. Accuracy of BP measurement	a. Blood pressure measurement training every 6 months
		b. Blood pressure measurement protocol
		c. Use automated and clinically validated BPMDs
	2. CVD risk assessment	a. Classify patients with existing CVD, diabetes, and chronic kidney disease as high CVD risk. Calculate the risk of CVD in the remaining patients
		b. Use statins and aspirin according to CVD risk level and history of CVD
Treatment	3. Standardized treatment protocol	a. Institutionalized Clinical Pathway
		b. Use antihypertensive medicine in fixed-dose combination
	4. Treatment intensification	a. Initiate pharmacological treatment without delays
		b. Intensify treatment until reaching BP control
Continuity of Care	5. Follow-up frequency	a. Monthly follow-up until reaching BP control
		b. Follow-up every 6 months in non-high CVD risk patients with controlled BP
		c. Follow-up every 3 months in high CVD risk patients with controlled BP
Delivery system	6. Team-based care and task shifting	a. Non-physician community health workers measure BP
		b. Nurses follow patients
		c. Nurses intensify pharmacological treatment according to the clinical pathway
	7. Medication refill frequency	a. Medication refill every 3 months
Performance evaluation	8. System for performance evaluation based on quality improvement methodology	a. System for monitoring and evaluation with monthly feedback

Adapted Ref 41. Copyright 2022 Pan American Health Organization. Published by Elsevier Ltd. This is an open-access article under the CCBY-NC-ND license(http://creativecommons.org/licenses/by-nc-nd/4.0/)

Then, the IG designed the HEARTS Maturity index to translate the eight key drivers into process indicators. Additionally, the Performance Index was designed to monitor the outcome indicators: program coverage and hypertension control among treated. The routine measurement and analysis of hypertension key drivers’ implementation is meant to identify areas where effectiveness can be maximized and prompt healthcare teams to devise corrective actions in response. Another notable feature of HEARTS’ quality improvement approach is it places the responsibility for the collection and analysis of data and the creation and implementation of corrective actions in primary health care centers. This lies at the heart of the strategy’s quality improvement methodology for targeting interventions and continuously improving program management [40].

### HEARTS Clinical Pathway

HEARTS Clinical Pathway is a decision support tool [38, 39]. It is intended for health systems that cover relatively large administrative areas or entire countries but can also be used by PHC centers even outside of HEARTS-implementing countries. It aims to drive the rapid adoption and scaling up of the 2021 WHO guideline on hypertension [31] while facilitating the systematic implementation of HEARTS hypertension control drivers [41]. It is the product of a broad consensus process among a multidisciplinary group of experts from 16 countries in the region coordinated by PAHO [40].

**Fig. 2:**
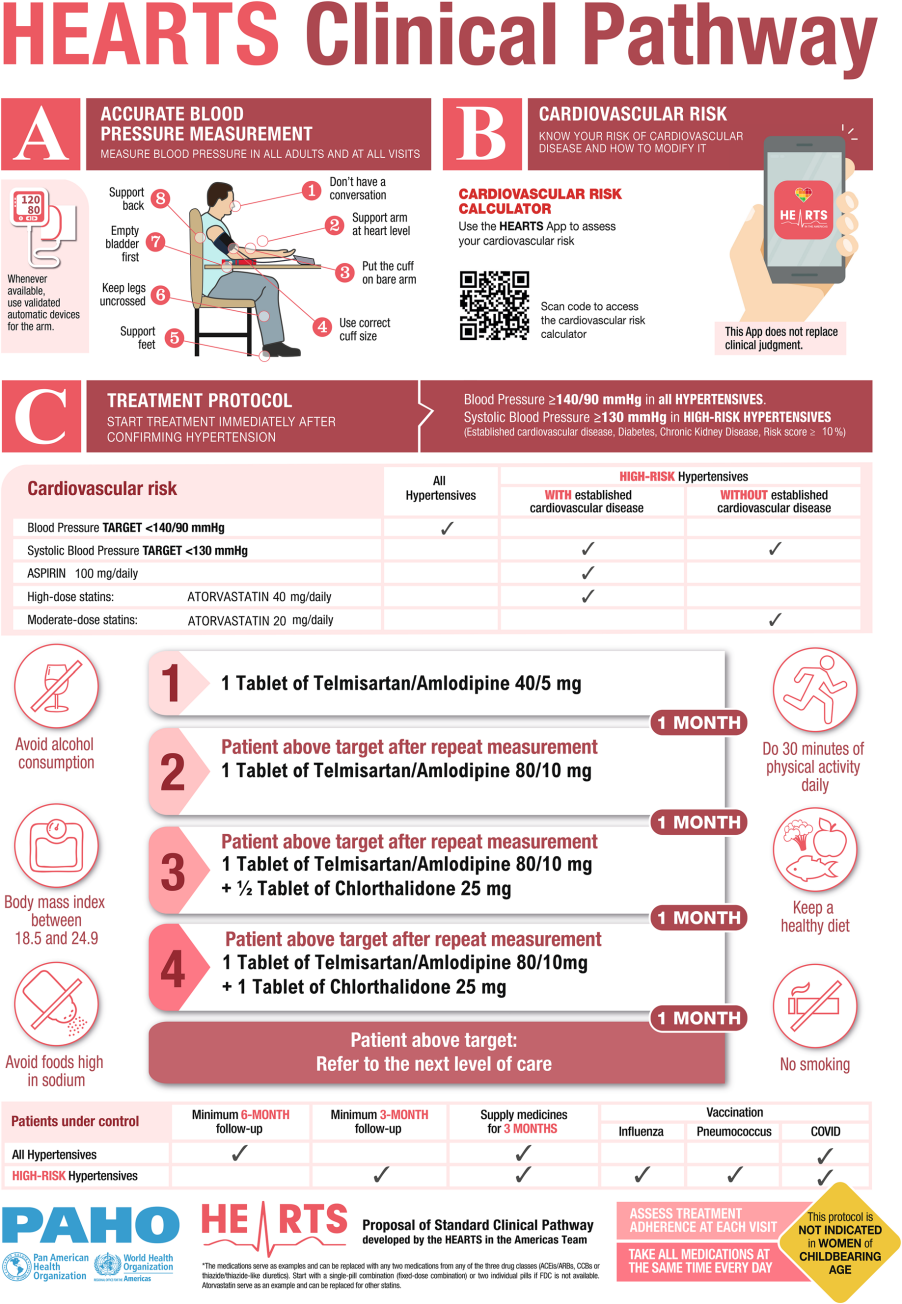
HEARTS Clinical Pathway (Adopted by Ref. 39). Adopted from Ref. 39. Copyright 2022 Pan American Health Organization. Published by Elsevier Ltd. This is an open-access article under the CC BY-NC-ND IGO license (http://creativecommons.org/licenses/by-nc-nd/3.0/igo/)

HEARTS Clinical Pathway comprises a set of comprehensive, structured, multidisciplinary, management plans that map the care pathway through the health system for people with hypertension and for high-risk hypertensives with and without established CVD. Most individuals with hypertension, across a wide spectrum of demographics such as age, race, ethnicity, socioeconomic status, geography, and culture, can be managed using a standardized clinical pathway. This represents the “patient rule.” A small percentage of individuals with hypertension will require an individualized approach, the “patient exception.” This standardized approach for most individuals with hypertension can reduce undesirable clinical variability outside the scope of evidence-based practice. It is simple, directive, actionable, feasible, and scalable. (Fig. 2). These characteristics also facilitate team-based care enabling multiple healthcare professionals, under supervision, to participate in BP measurement, medication titration, and clinical follow-up. Noteworthy, HEARTS Clinical Pathway is a powerful tool to inform patients about the optimal standard of care to reduce their risk of premature mortality or CVD and to encourage them to actively participate in their treatment.

HEARTS Clinical Pathway evolved rapidly from a standardized treatment protocol (STP) mainly focused on hypertension, as designed for Module E of the WHO HEARTS technical package [41]. Accordingly, the initial STP was transformed into the HEARTS Clinical Pathway [39] which comprises three main sections (a) hypertension detection and diagnosis, (b) CVD risk assessment and management, and (c) treatment and control. In addition, other important recommendations related to continuity of care, timely follow-up, and vaccination were included.

#### Hypertension Detection and Diagnosis

A population hypertension program that has the imperative of reducing the detection/awareness gap, which can reach 30–40% in some countries, must take advantage of each clinical encounter to measure BP in all adults accurately. This action can be complemented with community screening for cases in certain population groups that do not usually visit health services. In HEARTS in the Americas, BP measurement relies on a widely accepted clinical measurement protocol [42] and, whenever available, the use of automated, clinically validated blood pressure measuring devices (BPMDs) [43]. To promote accurate measurement of BP, PAHO is providing support for the training and certification of healthcare providers in the accurate measurement of BP [44] and has developed a regulatory framework model to be used as a tool by countries to move towards the exclusive use of validated BPMDs in PHC settings [45].

##### CVD Risk Assessment and Management

HEARTS in the Americas has developed a pragmatic approach, well aligned with the 2021 WHO hypertension guideline [31], to optimize CVD risk assessment and maximize the treatment impact. The clinical pathway recommends initiating pharmacologic anti-hypertensive treatment immediately after the diagnosis of hypertension. Waiting for laboratory results to complete the CVD risk estimation or to assess comorbidities could delay the initiation of treatment, especially in low-resource settings. Likewise, in a setting without resource constraints, the complexity of the diagnostic protocol could also be a factor that delays the start of treatment, negatively impacts retention, and overloads health systems.

Hypertension treatment and control is a critical component of the integrated management of CVD risk [36, 37, 46]. Thus, the HEARTS Clinical Pathway indication is expeditious: to classify as high risk of CVD, without using a risk score, but just by identifying established CVD (IHD, stroke or peripheral vascular disease), diabetes, and chronic kidney disease. Then, start pharmacologic treatment immediately in those with CVD or at high CVD risk when SBP ≥ 130 mmHg and rapidly up-titrate the dose and addition of other anti-hypertensive agents, if needed (every 2–4 weeks) until the patient’s BP is controlled (SBP < 130/80 mmHg). In addition, for patients with established CVD, add high-intensity statins and low-dose aspirin if there are no clinical contraindications. A similar therapeutic approach, but with moderate doses of statins and no aspirin, has been recommended for people at high risk but without established CVD [39, 47].

HEARTS in the Americas has developed a digital application (App) [48] to facilitate the implementation of this treatment approach. The CVD risk calculator, one of the components of the HEARTS App [49], based on the WHO CVD Risk Chart [50], allows the estimation of CVD risk and facilitates the treatment regimen based on the individual clinical profile and the specific clinical pathway adopted by each country.

##### Hypertension Treatment

An STP is the core of the HEARTS Clinical Pathway. Its purpose is to guide and optimize treatment delivery and reduce therapeutic inertia. Non-pharmacologic treatment with specific recommendations has a prominent position in the clinical pathway. However, it is not sufficient on its own and must be administered together with pharmacologic treatment [39]. It is simple, linear, with few titration steps, and non-discretionary, with specific medications and doses in each step. The treatment strategy is straightforward: the first step begins with the administration of two medications from complementary classes of agents, preferably in a fixed-dose combination (FDC), also called a single-pill combination, each at half maximum doses. Then, in the second step, it quickly progresses to the full dose of both medications. Afterward, in a third step, a third drug is added following the same intensification strategy: half a maximum dose followed by a full dose [51].

Ideal characteristics for individual drugs or FDCs selected to be included for the STP include high efficacy, additive/synergistic BP reduction when used in combination, mitigation of side effects of either or both individual agents, are tolerable, require minimal laboratory monitoring, have potential for wide availability and affordability, are once-daily dosed and are scored for easy titration [52]. For instance, the combination of a renin–angiotensin–aldosterone system inhibitor with a calcium channel blocker, in addition to its synergy, reduces the side effects of these medications, such as pedal edema, compared to when they are used in high doses and as monotherapy.

Improving the efficacy of medication treatment reduces hypertension-associated disease burden. In this regard, the delivery and distribution of affordable, effective single-pill combinations of two or three drugs is promising. [53]. Indeed, the combination of two drugs, in a single pill, has been one of the key interventions to explain the high and sustainable levels of hypertension control reached by the Kaiser Permanente program in California [18]. The combination of two drugs increases adherence and persistence, reduces pill burden and the time to reach BP control, and reduces costs associated with the number of visits and potential complications. In addition, two-medication combinations lower the BP equally in most patients, mitigating differences in medication efficacy issues associated with sex, age, race, or large multiethnic groups [51, 54].

The HEARTS Clinical Pathway is used to model clinical pathways in implementing countries. Indeed, high consistency and minimal clinical variability have been achieved in the clinical pathways in the participating countries [47]. Importantly, when a country sits down to develop its Clinical Pathway, the decision-makers are compelled to analyze and identify resource gaps in medications and validated BPMDs. This resource and gap analysis is a unique opportunity to proactively address these issues. When a country or region adopts its Clinical Pathway, an explicit commitment is established by the health authorities with the community and health providers to guarantee the availability, quality, and affordability of the selected medicines, and to seek timely alternatives if there are any disruptions in the supply chain. Finally, the HEARTS Clinical Pathway has also incorporated vaccination against influenza, pneumococcus, and COVID-19 as strategies for the prevention of cardiovascular complications in high-risk people. Because most patients should receive similar standardized care for hypertension, attention to known health disparities can be effectively addressed.

## Key Factors to Accelerate the Expansion of HEARTS

HEARTS in the Americas essentially face the same issues as other interventions designed to be implemented in primary care settings. First, there is often a lack of political will and sense of urgency in part because of many competing priorities. Second, there is a lack of understanding of the central role that hypertension plays as a cause of NCDs and in the management of other major chronic vascular diseases and even of other NCDs, especially in PHC settings. Third, there are well-known structural challenges in many primary care settings such as inadequate resources, high patient care burden, lack of sufficient equipment, staff shortages, and many others.

In this review, we will recap three essential issues for the implementation and scaling up of HEARTS, (a) medicines, (b) team-based care, and (c) systems for monitoring and evaluation. Although we will focus on hypertension, these factors have the potential to strengthen primary care and, ultimately, help transform health systems.

### Essentials for Implementing and Scaling HEARTS

#### To Be, or Not to Be

Lowering the price and increasing the quality and availability of medicines is a key priority for scaling up hypertension control and making hypertension programs sustainable. What we have learned from implementing HEARTS in the Americas is that access to quality medicines is a major barrier to treatment [3].

A recent evaluation conducted by PAHO in six HEARTS-implementing countries found that the availability of medicines for hypertension is not an issue of patents. Failures stem in part from the lack of updates to the national essential medicines list (EMLn). EMLn provides an incentive for the industry to process the corresponding sanitary registry for commercialization. Consequently, in countries with few registries for a specific medicine, prices tend to be higher. Likewise, in countries where multiple organizations buy small volumes, medicines are more expensive. The Strategic Fund of PAHO is a regional mechanism for pooled procurement that can bring together the demand for preferred medicines from various countries to achieve lower purchase prices than the price paid by most countries for the same products [55].

An STP, supported by a core set of medicines, is key to improving access by reducing prices and ensuring reliable supply by simplifying the supply chain. While pharmaceutical companies have prioritized the production of many medications across the main anti-hypertensive classes, the HEARTS pharmacologic strategy focuses on a limited number of drugs and related combinations in a single pill. Indeed, a fragmented market may serve to fracture demand across many products, diminish the purchaser’s power to negotiate lower prices and, in some cases, reduce suppliers’ economies of scale, which increases production costs. STPs will also facilitate more accurate and long-term forecasting of product needs both at local and national levels [56].

FDCs in a single pill, included in the WHO EML since 2019 [57], have emerged as an important addition to reducing prices. The use of FDCs can further simplify procurement and supply chains and have the potential to minimize strain on under-resourced supply chains by reducing the number of products and related transactions in a given supply chain. In addition, the smaller packaging footprint of FDCs versus their separate components may reduce costs related to freight and decrease storage space [56].

##### Come Together

Team-based care with task-shifting is one of the main components of the HEARTS approach to improve access, quality, and continuity of care [57]. Tasks include providing care to patients, independent prescribing, counseling, and education, with comparable quality of care. Pharmacists and nurses can potentially undertake substantially expanded roles to support physicians in the PHC in response to the changing health service demand. This requires the optimization of organization systems, enhanced education programs, and engagement with all stakeholders to ensure health systems strengthening [58, 59].

The 2021 WHO Guideline for hypertension recommended that pharmacological treatment of hypertension can be provided by nonphysician professionals, such as pharmacists and nurses, with proper training, and prescribing authority, based on specific management protocols under physician oversight [31]. Indeed, team-based care is superior to the traditional physician-focused model [60, 61]. For example, a multicomponent intervention centered on proactive home visits by trained community health workers, who were linked with existing public health care infrastructure, led to a greater reduction in BP than usual care among adults with hypertension in rural communities in Bangladesh, Pakistan, and Sri Lanka [62]. Further, a cluster randomized clinical trial in China has shown that a strategy based on care provided by community health workers, using an STP, was more effective in reducing BP compared to the usual model. Remarkably, a significant reduction of 23% in the incidence of myocardial infarction and 34% in the incidence of stroke was observed. These figures translated into a 30% reduction in mortality from CVD causes and a 15% reduction in all-cause mortality [63••].

Evidence in favor of a team-based care approach is solid and growing. However, in general, this approach has not found the receptivity it deserves in LAC. For instance, drug titration by non-physician health workers, such as nurses and pharmacists, even under the supervision of a physician and guidance of an approved treatment protocol, remains an underused care delivery practice in most countries. Traditions, culture, and normative elements seem to coexist and emerge as barriers that prevent the development of a more effective and efficient system [47].

##### No Data, No Progress

A universal electronic medical record (EMR), with a patient portal, should be one of the standards of clinical care. It is essential for individual monitoring, identifying those needing further care, performance evaluation, and a way to engage patients and their families in their long-term care. Additionally, and linked with the EMR, regularly reporting a core set of standardized metrics for hypertension allows care teams to review and understand their performance and promptly correct any deviations from established performance standards over time. The standardization across the implementing sites allows comparison across similar programs as well as learning from high-performing programs. Selecting a quality metric that can be measured easily and shared widely and regularly is more critical than selecting more complex indicators [34].

However, the precariousness of health information systems in most implementing countries is a major issue for scaling up HEARTS in the Americas. In fact, from a programmatic perspective, only PHC facilities that regularly report data are considered HEARTS implementing centers. For instance, the majority of implementing countries do not have a system that allows the monitoring of patients or evaluation of the program. Some have paper-based systems that are only marginally effective. Most have an information system, top-down oriented, designed for administrative information without the capacity to collect clinical, process, or outcome data. Most countries have multiple small and fragmented health information systems that are not interconnected [64, 65].

Accordingly, HEARTS in the Americas responded, first, with a standardized methodology and a list of indicators to guide and assess the progress of the program [66] and then with a digital platform for monitoring and evaluation [65]. This new platform, based on an open-source software known as DHIS2 [67], focuses on aggregated data entry from PHC facilities, timely reporting of data, and use of data to better respond to the needs of the population in their catchment areas. Undoubtedly, there are challenges beyond infrastructure. These include the lack of an institutionalized culture based on quality improvement, lack of incentives, and resistance to or unfavorable perception of evaluation or clinical audit. Also, contributory is the lack of policies compelling health authorities to share health outcomes and population management data with the communities they serve. Beyond the challenges, the new platform can support program implementation, reveal structural and managerial limitations and care gaps, uncover hidden disparities, and lead to favorable changes at different levels of the health system [64, 65].

## Cases of Successful Implementation

HEARTS in the Americas has progressively become a vibrant initiative, with in-depth regional scope. Although HEARTS does not advance with the same speed or with the same depth in all countries, it is moving forward and beginning to show its first results. Indeed, there are some very inspiring cases.

Chile and Cuba, with a long tradition of strong primary care systems, have made rapid progress in the implementation of HEARTS. Both have created robust governance and accountability mechanisms for this initiative and the scale-up in primary care has contributed to the improvement of the quality of services provided in the post-pandemic recovery stage. HEARTS Clinical Pathways are nationally endorsed with guarantees of access to medicines and services. The use of clinically validated BPMDs has grown rapidly, replacing aneroid manometers. In these countries, the absolute number of people undergoing treatment and the hypertension control among those treated have increased [68–70]. In Chile, the HEARTS model is proving to be superior to traditional care: better control among those treated (65% vs. 40%), shorter time to reach a BP level below 140/90 mmHg (31 vs. 92 days), and better adherence and persistence after one year of treatment (71% and 20%, respectively) have been demonstrated [71]. In Cuba, with limited pharmacological treatment options, 78% of the hypertensive population in the catchment area was documented to be enrolled in the program, with 59% facility-based BP control in the 22 PHCs that began implementing the model [72].

Saint Lucia and Trinidad and Tobago lead the implementation of HEARTS in the Caribbean, a sub-region that has enthusiastically committed to the implementation of this care approach. Both countries have created strong, effective, and inter-programmatic governance mechanisms, and have extended the model to all their PHC facilities. They also stand out for their high-quality clinical pathways and the use of validated automated BPMDs. Saint Lucia is one of the few countries in the world that starts the treatment of hypertension from the first step with the use of an FDC. In both countries, HEARTS has been very well received by patients and providers and the first evaluation results indicate a sustained increase in the program coverage [73, 74].

Argentina, Mexico, and Peru have established their clinical pathways with the involvement and support of all stakeholders, including health entities, academia, and professional societies. Thus, the implementation of the HEARTS Clinical Pathway is significantly influencing the decision-making processes for the selection, budgeting, and purchase of antihypertensive drugs and BPMDs. It has also favored a change in the focus of pharmacological treatment and the scope of practice with greater participation in primary care. In the province of La Rioja, Argentina, HEARTS implementation increased the treatment rates and the use of combined treatment after 18 months of implementation in a province serving one of the most vulnerable communities [75]. In Peru, HEARTS has been progressively spread and showed an increase in the levels of hypertension coverage and control [76]. In Mexico, implementation is leading to important changes in information systems with a focus on strengthening the primary care network [77]. The model in Mexico is not only improving health outcomes related to hypertension control but is also enabling proposals for other effective systematic evidence-based implementation interventions that can reduce the costs of implementing the programs [78].

Many more stories demonstrate the penetration of HEARTS and its positive influence in the region. Among the most relevant are those related to training and education. For example, more than three-quarters of a million healthcare professionals, mainly PHC teams implementing HEARTS, and health sciences students, have taken HEARTS virtual courses hosted by the PAHO virtual campus of Public Health [3]. In the Dominican Republic, the training and learning materials produced by HEARTS have begun to be used by universities in undergraduate teaching. In Chile, HEARTS certificates are beginning to be recognized as valid for continuing medical education. In Bolivia and Mexico, primary healthcare teams, including thousands of nurses, have been instructed to use HEARTS training and learning materials.

## Final Outlook

The conversation around hypertension control in LMICs, and high-income countries, must go beyond pathophysiology, individualized approach, monotherapy, clinical trials, and clinical guidelines. The new paradigm, without which great advances in the science of hypertension cannot be realized, is the implementation of standardized, population, and evidence-based recommendations through public health programs designed to have a high impact, effectively and efficiently. This issue is poorly addressed and generally missing in the current hypertension landscape.

In this review, we have summarized the epidemiological situation that justifies the urgency and priority for implementing HEARTS in the Americas, the rationale and characteristics of its main components, the key factors to catalyze and scale up its implementation, and finally, we highlighted the success stories that realize the transformative potential of HEARTS.

HEARTS in the Americas is helping to energize the changes that PHC systems need to respond to the shortcomings of a traditional hypertension care model that was functioning poorly, even before COVID-19. That old model, poorly prioritized, physician/specialist-centered, based on long and complex guidelines, operating with outdated medicines and non-clinically validated manometers, without monitoring or feedback, has failed to improve hypertension control beyond the meager third of all hypertensives in most countries worldwide. Undoubtedly, when attempting to introduce a new paradigm, HEARTS faces many challenges. These challenges encompass not only profound and fundamental structural issues relating to health systems but also extend to cultural barriers, entrenched traditions, established norms, old schools of thought, and power dynamics.

Beyond those challenges, the number of countries and PHC facilities implementing the HEARTS model has grown exponentially since 2017. Its first programmatic target will be set by 2025 when most countries have committed to scaling the model to their entire PHC network. Although clinically important (“hard”) outcomes such as reduction in stroke and IHD may still take some time to accrue, the increase in hypertension coverage and control in primary health care settings compared with the traditional model is promising and confirms that the set of interventions under the HEARTS umbrella is feasible and acceptable to communities, patients, providers, decision-makers, and funders. The development and expansion of telemedicine and home BP monitoring can further improve access and continuity of care, the engagement of people with hypertension, and ultimately the quality and efficiency of care programs.

In summary, the reduction of premature mortality from NCDs is highly dependent on the prevention and treatment of CVD and especially dependent on improved hypertension management. Substantial reductions in the burden of CVD could be achieved relatively quickly if innovative interventions such as those recommended by HEARTS were systematically and widely implemented. Therefore, scaling up effective treatment for hypertension and optimization of CVD risk management is a pragmatic way to accelerate the reduction of CVD mortality while strengthening primary healthcare systems to respond effectively, with quality, and equitably, to the challenge of NCDs, not only in LMICs but in all communities globally.
